# Innovative Approaches in the Battle Against Cancer Recurrence: Novel Strategies to Combat Dormant Disseminated Tumor Cells

**DOI:** 10.3389/fonc.2021.659963

**Published:** 2021-04-27

**Authors:** Scott Sauer, Damon R. Reed, Michael Ihnat, Robert E. Hurst, David Warshawsky, Dalit Barkan

**Affiliations:** ^1^ Vuja De Sciences Inc., Hoboken, NJ, United States; ^2^ Department of Individualized Cancer Management, H. Lee Moffitt Cancer Center and Research Institute, Tampa, FL, United States; ^3^ Cancer Biology and Evolution Program, H. Lee Moffitt Cancer Center and Research Institute, Tampa, FL, United States; ^4^ Adolescent and Young Adult Program, H. Lee Moffitt Cancer Center and Research Institute, Tampa, FL, United States; ^5^ Department of Pharmaceutical Sciences, College of Pharmacy, Oklahoma University Health Sciences Center, Oklahoma City, OK, United States; ^6^ Department of Human Biology and Medical Sciences, University of Haifa, Haifa, Israel

**Keywords:** tumor dormancy, metastasis, disseminated tumor cells, cancer recurrence, tumor microenvironment, immune evasion and clinical trials

## Abstract

Cancer recurrence remains a great fear for many cancer survivors following their initial, apparently successful, therapy. Despite significant improvement in the overall survival of many types of cancer, metastasis accounts for ~90% of all cancer mortality. There is a growing understanding that future therapeutic practices must accommodate this unmet medical need in preventing metastatic recurrence. Accumulating evidence supports dormant disseminated tumor cells (DTCs) as a source of cancer recurrence and recognizes the need for novel strategies to target these tumor cells. This review presents strategies to target dormant quiescent DTCs that reside at secondary sites. These strategies aim to prevent recurrence by maintaining dormant DTCs at bay, or eradicating them. Various approaches are presented, including: reinforcing the niche where dormant DTCs reside in order to keep dormant DTCs at bay; promoting cell intrinsic mechanisms to induce dormancy; preventing the engagement of dormant DTCs with their supportive niche in order to prevent their reactivation; targeting cell-intrinsic mechanisms mediating long-term survival of dormant DTCs; sensitizing dormant DTCs to chemotherapy treatments; and, inhibiting the immune evasion of dormant DTCs, leading to their demise. Various therapeutic approaches, some of which utilize drugs that are already approved, or have been tested in clinical trials and may be considered for repurposing, will be discussed. In addition, clinical evidence for the presence of dormant DTCs will be reviewed, along with potential prognostic biomarkers to enable the identification and stratification of patients who are at high risk of recurrence, and who could benefit from novel dormant DTCs targeting therapies. Finally, we will address the shortcomings of current trial designs for determining activity against dormant DTCs and provide novel approaches.

## Introduction

Recent years have seen great strides in the treatment of primary tumors, as well as in treating overt metastatic tumors. Nonetheless, the main cause of mortality of cancer patients remains metastasis and recurrence. Metastasis, the spread of tumor cells from the primary site to secondary organs accounts for 67-90% of all cancer mortality (1, 2). Despite significant improvement in the overall survival of many types of cancer due to earlier detection and newer therapies, recurrence years and decades after curative surgery and standard of care chemotherapy and targeted therapy (3–5) still looms as the major unmet medical need.

Some cancers are more notorious than others for delayed recurrence. These highly recurrent tumors include kidney cancer, acute myeloid leukemia (AML), non-small cell lung cancer (NSCLC), melanoma, prostate cancer, ovarian cancer, breast cancer and osteosarcoma. Kidney cancer and AML exhibit low single/double-digit recurrence rate (6–8), while many other cancers have a very high rate of locoregional and distant recurrence despite treatment with standard of care (**Table 1**).

**Table 1:** Estimated Recurrence Rate of Various Cancers.

Cancer	Recurrence Rate
Breast	30% distant recurrence (9, 10)
Glioblastoma	~100% (11)
Prostate	20-30% (12)
Leukemia, childhood AML	9-29% (6)
NSCLC	30-50% locoregional or distant recurrence (13, 14)
Osteosarcoma	30-40% (15, 16)
Ovarian	85% (17)
Pancreatic	36% within 1 year of curative surgery 38% local recurrence & 46% distant metastasis after adjuvant chemotherapy (18, 19).
Melanoma	50% of all patients treated for melanoma will have a recurrence. Of these recurrences, ~50% will be in the regional lymph nodes, 20% will be local recurrences, and 30% will arise at distant sites (20)

Lung cancer is the leading cause of cancer-related death with NSCLC accounting for ~90% of new cases (21). Even when curative surgery is performed, 30-50% of NSCLC patients develop locoregional or distant recurrence (22, 23). Melanoma can recur in 50% of the patients (20) sometimes a decade or more following removal of the primary tumor (24). Prostate cancer, which is a slow growing tumor, nonetheless shows biochemical recurrence (increasing PSA) even in low risk patients beginning some 4 years following therapy with curative intent in some 25% of patients (12). In ovarian cancer, an estimated 85% of patients who achieve full remission after initial treatment (surgery and adjuvant chemotherapy) have a recurrence, with median survival between 12-24 months after recurrence (17, 25). Breast cancer is the most common form of cancer in US women after skin cancer, with over 275,000 cases estimated for 2020. Much progress has been made in the treatment of primary breast cancer, particularly when there is a known mutation or overexpression that can be directly targeted (e.g. PI3K, CDK4/6, PARP, PD-L1) or with hormone receptor-positive disease (e.g. ER+, PR+, HER2+) (26). Even with the wealth of treatment options, around 30% of all breast cancer patients with no detectable disease post-treatment present with recurrence on follow-up (9). Osteosarcoma (OS) is the most common type of primary bone tumor accounting for 30-80% of skeletal sarcomas, and it occurs primarily in adolescence (27). Even with aggressive treatment including limb-salvage surgery and chemotherapy, the rate of recurrence in patients presenting with non-metastatic OS is 30-40% (15, 16). Taken together, despite major advances in the treatment of primary tumors, almost all cancer-related mortality is due to recurrence and metastasis and many of the most common cancers have a significant propensity for delayed recurrence (28).

While metastasis and recurrence are the main cause of mortality in cancer patients, the mechanisms underlying metastatic recurrence years and decades after initial treatments are just beginning to unravel. Recurrence due to metastatic spread begins as a multi-step process that can take months or years until it becomes detectable. Recent studies have shown that although the probability of metastasis increases with the size of the primary tumor, cancer cells nonetheless leave primary tumors early (29–32) and settle in distant tissues to become disseminated tumor cells (DTCs).

Once cancer cells arrive at their new and foreign microenvironment (‘non-permissive soil’) they face several fates. The majority of them will undergo apoptosis and thus will meet their demise. Those that successfully launch adaptive and survival programs will enter a dormant state. Some dormant DTCs may reside as single solitary quiescent cells (cellular dormancy) and/or as small clusters of quiescent cells. Others may reside as small indolent micrometastases where cellular proliferation is balanced by apoptosis (33–36). These indolent micrometastases remain dormant due to either lack of angiogenic signals (angiogenic dormancy) that promote recruitment of the vasculature needed to nourish the micrometastatic tumor (35, 36) and/or involvement of the adaptive immune system (immune-mediated dormancy) (37). To date, there are no imaging moieties to detect dormant quiescent and indolent micrometastases in patients. Furthermore, due to their quiescence, these dormant DTCs are resistant to classical anticancer therapy that relies on rapidly dividing cells to exert their effect. Therefore, dormant DTCs linger in the body as ticking time bombs and eliminating or keeping such cells at bay may prevent deadly metastatic relapse.

Cell-intrinsic mechanisms governing DTC dormancy and escape from dormancy are influenced by signals arising at their foreign niche. Accumulating evidence in the literature attributes dormancy and survival of residing DTCs and their reactivation to the intricate cross- talk with their ‘non-permissive’ or their ‘permissive’ niche, respectively (38–43). Hence, we can postulate that by manipulating the microenvironment and/or cell-intrinsic mechanisms we may either be able to eradicate dormant DTCs, maintain dormant DTCs at bay, or prevent their transition to overt metastases.

This review will focus on potential strategies, mechanisms and drugs to be considered for targeting quiescent dormant DTCs by manipulating their microenvironment and or their cell- intrinsic mechanisms. We will initially present clinical evidence for the presence of dormant DTCs. Potential biomarkers to enable the identification and stratification of patients who are at high risk of recurrence will also be discussed. Finally, we will address the shortcoming of current clinical trial designs for demonstrating activity against dormant DTCs, either quiescent or indolent, and provide novel trial designs.

## Clinical Evidence of Tumor Dormancy and Recurrence

Demonstrating the presence of dormant DTCs and their subsequent progression in clinical settings has been challenging. However, advances in detection have provided new information and a growing body of evidence in the clinic to support the idea of early dissemination of tumor cells from the primary site, followed by subsequent dormancy and late recurrence.

One clinical example that supports tumor dormancy is late-stage recurrence. Recurrence of a tumor after more than 5-years remission is in line with an initial dormancy period followed by reactivation and outgrowth. In support of this idea, it was found that breast cancer patients who do not have detectable disease can have circulating tumor cells (CTCs) found in their blood 20+ years after initial treatment (44). The inability of tumor cells to survive decades in the blood supports the hypothesis that these cells are being shed from undetectable DTC populations that have remained dormant for years. Minimal residual disease, or tumor cells that remain in the body after initial treatment, is typically undetectable at the primary site but can be found in the circulation, bone marrow or other organs prone to recurrence (e.g. lungs, liver) (45). Even before late-stage recurrence, these cells can be identified and characterized from blood or bone marrow and have been found to upregulate programs that promote dormancy, survival and progression. One feature observed in bone marrow-resident DTCs is a decrease in proliferation markers Ki67 and proliferating cell nuclear antigen (PCNA), which support the idea that these cells are quiescent and thus less susceptible to cytotoxic chemotherapies (29, 45). Additionally, multiple pathways implicated in dormancy and recurrence in laboratory models are overexpressed in recurrent tumors. In stomach cancer, urokinase-type plasminogen activator receptor (uPAR) was upregulated in patients whose cancer recurred, while low levels of uPAR correlated with longer disease-free periods and survival (46). This same trend was observed with HER2/ERBB2 overexpression in disseminated breast cancer tissue correlating with worse outcomes (47).

Another clinical observation in support of early metastasis and subsequent dormancy can be found in cases of unknown-primary carcinoma (UPC). UPC accounts for ~5% of metastatic cancer cases, and while some cases are later identified after more thorough evaluation, 30% of patients never have a primary site identified (48). These unexplained cases of UPC are believed to have formed from DTCs from a primary tumor that regressed and could no longer be observed (Riethmüller and Klein 2001). Detection of UPC in cases where the primary site is never determined highlight the lack of clinical understanding for tumor cell dissemination.

One of the best pieces of evidence for dormant DTCs that can recur after a long period of quiescence is seen in cases where patients develop tumors after organ transplantation. Accidental transmission of tumors from tissue transplants derived from seemingly cancer-free cadavers was first reported in the case of kidney transplants (48). In one of the earliest reported instances, metastatic squamous cell carcinoma occurred in a patient 8 months after a kidney transplant from a donor later found to have larynx carcinoma (Tissue Transplantation Still Vexes 1965). Another instance of kidney transplant-related cancer saw the patient remain disease-free for 3 years before being diagnosed with metastatic liver cancer. Surprisingly, one study found that of 164 patients receiving organ donations from donors eventually diagnosed with cancer, 44% developed tumors with the majority of those cases related to the tumors of the original donors (49). In a later study, a heart donor was diagnosed with prostate cancer post-mortem, and 10 months after transplant the recipient was diagnosed with multiple metastatic lesions in the spine, sacrum and ribs. Genetic analysis of the lesions indisputably matched with the donor’s prostate and kidney, providing evidence at the molecular level that the tumor was derived from quiescent prostate DTCs in the donor’s heart (50). All of this clinical evidence provides direct support that tumor dissemination can be an early event and tumor dormancy allows these cells to evade therapy and the immune system and detection for many years, even decades, before leading to recurrence.

## Putting DTCs Under the Dormancy Spell

Clinical data demonstrate the presence and persistence of dormant DTCs years and even decades after treatment. In some cases, these DTCs will remain dormant without relapsing. Hence, unraveling the mechanisms responsible for their long term ‘hibernation’ may set the premise to develop novel therapeutic strategies to prevent cancer from recurring by keeping them dormant indefinitely.

### Reinforcing the Dormant Niche

**Figure 1:**
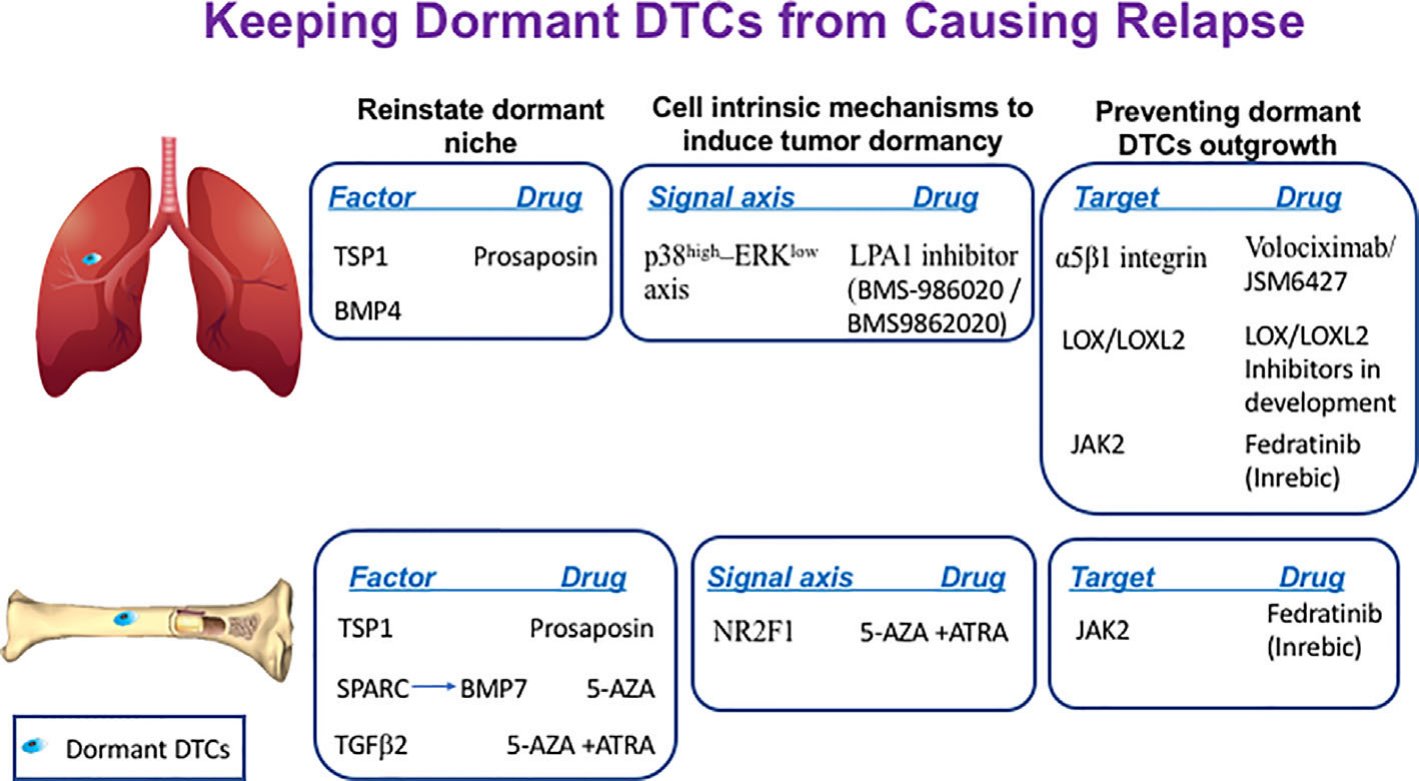
Keeping dormant DTCs from causing recurrence. The following scheme illustrates the different sites where dormant DTCs reside and potential factors, signaling axis or targets that can be manipulated by the indicated drugs to maintain their long-term quiescence by either reinstating the dormant niche, inducing cell-intrinsic dormancy mechanisms and/or preventing dormant DTCs engagement with their ‘permissive niche’.

Several restrictive signals have been described in the bone marrow (BM) and lung that maintain DTCs originating from breast, prostate, head and neck squamous carcinoma and multiple myeloma cells in their quiescent state (**Figure 1**). Therefore, the BM is seen as a sanctuary site for DTCs. Growth arrest-specific 6 (GAS6) (51), Wnt5α (52), leukemia inhibitory factor (LIF) (53) and TGF-β family members such as bone morphogenic protein 7 (BMP7) (54) and transforming growth factor beta 2 (TGFβ2) (55, 56) were shown to exert quiescence of DTCs at the BM niche, whereas, bone morphogenic protein 4 (BMP4) was shown to promote tumor dormancy in the lung (57). Hence, theoretically we can postulate that DTCs can be maintained quiescent for prolonged periods of time by introducing the restrictive mediators that constitute the dormant niche (42).

SPARC, also known as osteonectin, was shown to regulate tumor dormancy of prostate cancer cells by promoting the expression of BMP7 in BM stromal cells. SPARC was shown to be epigenetically silenced in aggressive cells by promoter methylation whereas treatment of prostate cancer cells with the DNA demethylating agent 5-azacytidine (5-AZA) or with the COX2 inhibitor NS398 could restore SPARC expression in malignant prostate cancer cells. This in turn promoted BMP7 expression in BM stromal cells leading to dormancy of prostate cancer cells (58). Hence, reinstating SPARC expression in bone DTCs by treatment with either 5-AZA or with COX2 inhibitor NS398 may offer a therapeutic window to treat recurrent prostate cancer disease (**Figure 1**).

Another component that may be reinforced is thrombospondin-1 (TSP1), which is found in the suppressive BM and the lung niches. TSP1 secreted by stable microvasculature or by recruited BM-derived myeloid cells was shown to induce tumor dormancy of breast cancer cells at the perivascular niche (PVN) in the BM (59) and prevent metastatic outgrowth of breast and prostate cancer cells in the lungs (60). Given that TSP1 is a large protein, it is not feasible to consider it as a potential treatment. However, TSP1 can be induced by prosaposin. Indeed, administration of the TSP1 mimetic peptide prosaposin (DWLPK) was shown previously to induce TSP1 in BM-derived myeloid cells recruited to the lungs. The latter in turn assembled a metastasis-suppressive microenvironment (60) (**Figure 1**).

Hence, the approach of inducing natural factors of the suppressive niche such as TSP1 and or SPARC is very appealing. However, this approach will require continuous treatment to ensure indefinite quiescence of the residing dormant DTCs and thus may lead to potential toxicities. Furthermore, TSP1 has been also reported to exert opposing effects which can be also attributed to multiple receptors that mediate TSP1 signaling (61). Likewise, SPARC was reported to have context and tumor dependent impacts on tumor progression (62). Therefore, we must consider TSP1/SPARC’s multiple effects along with the type of tumor and stage of the disease if we want to consider it as a future preventive treatment for cancer recurrence. Another important aspect that should be taken into account when striving to reinforce the dormant niche with TSP1 or other suppressive constituents such as TGFβ family members, is the role they also play in the immune evasion of DTCs (61, 63).

Therefore, it may be more practical to induce common cell-intrinsic mechanisms converging from the different microenvironmental cues comprising the dormant niches.

### Promoting Cell-Intrinsic Mechanisms to Induce Tumor Dormancy

Pioneering work by Aguirre-Ghiso and colleagues identified p38-MAPK (mitogen-activated protein kinase) induction vs. reduction in ERK (extracellular signal-regulated kinase)-MAPK signaling (p38^high^–ERK^low^ signaling axis) as hallmark of tumor dormancy in several types of tumors (35, 64–66). Interestingly, Debio-0719, an inhibitor of lysophosphatidic acid receptor 1 (LPA1), was shown to induce tumor dormancy of triple-negative breast cancer (TNBC) cells at distant organs by inducing the p38^high^–ERK^low^ signaling axis (67). Though this inhibitor is still at the preclinical stage, some other inhibitors of LPA1 are currently being tested in clinical trials for fibrosis. These inhibitors include LPA1 inhibitor BMS986202 (previously AM152) and BMS-986020 [reviewed in (68)]. Therefore, potential use of these inhibitors in TNBCs patients as means to maintain residual disease at halt should be considered for future investigations (**Figure 1**).

Orphan nuclear receptor NR2F1 was shown to be a critical node for dormancy induction in head and neck squamous cell carcinoma (HNSCC) and in DTCs of prostate cancer patients (69). NR2F1 was found to control tumor cell dormancy *via* SOX9 and RARβ-driven quiescence programs (70). Furthermore, combining 5-AZA with trans-retinoic acid (ATRA), reinstated in part the NR2F1-induced dormancy program in HNSCC (70) and induced TGFβ2. TGFβ2 is a BM-derived factor shown previously to impose dormancy in HNSCC and in prostate cancer cells (55, 56). Hence, this combination can induce both dormancy programs and may also contribute to the formation of dormant niche (**Figure 1**).

### Preventing the Reawakening of Dormant DTCs by Targeting Their Crosstalk With Their Supportive Niche

The microenvironment of the metastatic niche (34, 36, 40, 71) and its remodeling (35, 38) plays a fundamental role in dictating the fate of residing dormant DTCs by inducing cell-intrinsic mechanisms culminating in the escape from tumor dormancy (39) (**Figure 1**).

Several reports implicated the role of chronic inflammation (72–74) and/or fibrosis (75, 76) as instigators of DTCs awakening. Fibrosis occurs due to a dysregulated wound healing response. Formation of a fibrotic-like milieu in the lung enriched with type I collagen (Col-I) and fibronectin was part of the tumor ‘permissive’ microenvironment to support dormant mammary DTCs outgrowth (75). Utilizing a 3D model system to study tumor dormancy (77, 78) it was demonstrated that fibronectin and Col-I induced beta 1 integrin (Intβ1) downstream signaling in dormant mammary cells *via* activation of focal adhesion kinase (FAK) by Src. This activation resulted in downstream activation of ERK, which in turn induced phosphorylation of myosin light chain (MLC) by myosin light chain kinase (MLCK), culminating in F-actin stress fiber organization and transition from quiescence to proliferation. Inhibition of MLCK activation (75, 77) and or Intβ1 expression (75) prevented dormant DTCs outgrowth *in vitro* and *in vivo*. Similarly, sustained lung inflammation caused by tobacco smoke exposure or nasal instillation of lipopolysaccharide (LPS) induced the outgrowth of dormant DTCs in the lungs by formation of neutrophil extracellular traps (NETs), which in turn lead to cleavage of laminin-111 by NET-derived elastase and MMP-9 and induction of the Intβ1/Src/FAK/MLCK axis (79). In addition, prostate cancer patient-derived xenograft lines were shown to transition from their dormant state once they engaged with the BM stoma by constitutively activating MLCK (80). Hence, MLCK may serve as potential target to prevent awakening of the dormant DTCs. However, given that MLCK is widely expressed in many normal tissues and in smooth muscle cells, presents a clinical challenge that may require the exploration of other avenues to inhibit MLCK activation in dormant DTCs. One such indirect approach to consider is inhibiting Intβ1 activation.

Notably, several studies highlighted the potential role of Intβ1 activation in regulating the dormant to proliferative switch (81, 82). Previous work reported how the cross talk between Intβ1 and the urokinase receptor can dictate the fate of dormant breast and head and neck cancer cells (83, 84). Intβ1 partners with α subunits to form 12 potential integrin receptors, which bind to extracellular matrix (ECM) molecules such as collagens, laminin, and fibronectin (85).

Indeed, several pre-clinical studies successfully inhibited Intβ1 activity, including α5β1 [reviewed in (86)] which binds the ECM protein fibronectin. In the clinical settings, the anti-α5β1 integrin antibody, volociximab, in combination with carboplatin and paclitaxel demonstrated some encouraging preliminary results in a Phase Ib clinical trial in advanced non-small-cell lung carcinoma (87). Interestingly, JSM6427 a small molecule inhibitor of α5β1 integrin was evaluated in a Phase I clinical trial for the treatment of age-related macular degeneration. It warrants further investigation whether JSM6427 could also be effective in preventing the awakening of dormant DTCs given its mode of action (http://clinicaltrials.gov/ct2/show/NCT00536016) (**Figure 1**). JSM6427 was shown to inhibit attachment of human retinal pigment epithelium cell (RPE) to fibronectin. This in turn promoted quiescence and cortical organization of the cytoskeleton of RPE. Similarly, inhibition of Intβ1 binding to fibronectin prevented the awakening of dormant mammary cancer cells resulting in their cortical F-actin organization reminiscent of the cytoskeletal organization of dormant DTCs (75, 77).

Therefore, if repurposing JSM6427 to treat cancer patients is being considered, the clinical regimen by which this drug will be administered must also be considered. In light of initial studies demonstrating Intβ1 plays a perquisite role in the reactivation of dormant DTCs [reviewed in (88)] while having no significant impact on actively proliferating metastases (75). Hence, these findings should be considered when designing future clinical trials with α5β1 integrin inhibitors. Changing the therapeutic paradigm of cancer therapy to a preventive treatment targeting early dormant DTCs rather than the current strategies aimed at treating patients with already advanced disease should be considered. Moreover, given that fibronectin and Col-I are part of the fibrotic milieu, preventing engagement of residual cells with such a supportive milieu after local surgery seems crucial.

Indeed, surgical trauma induces local and systemic inflammatory responses that can also contribute to the accelerated growth of residual and micrometastatic disease (89–91). Hence, intervention with inhibitors for α5β1 integrin should be pre-operative and immediately after surgery (post-operative).

Another receptor shown to interact with Col-I at the permissive site is DDR1. Col-I was shown to boost the association of DDR1 with TM4SF1, which, in turn, induced non-canonical signalling through the JAK2/STAT3 axis in dormant breast cancer cells leading to their outgrowth at multiple organ sites (92). Given that development of DDR1 inhibitors are at their infancy, a selective oral JAK2 inhibitor such as fedratinib (Inrebic^®^), recently approved by the FDA for the treatment of myeloproliferative neoplasm-associated myelofibrosis (93) may be considered as a potential drug to be tested in a preclinical setting (**Figure 1**).

In addition to preventing the engagement of dormant DTCs with their permissive niche and inhibiting cell-intrinsic mechanisms induced by signals arising at this niche, inhibiting the formation of such a permissive niche could represent a viable approach. For instance, inhibiting the cross-linking of Col-I by either lysyl oxidase (LOX) or lysyl oxidase like 2 (LOXL2), could prevent formation of the fibrotic milieu. Indeed, it was shown that LOX and/or LOXL2 inhibition significantly decreased pulmonary metastatic burden (94, 95). Furthermore, LOXL2 was shown recently to exert a cell autonomous role in the emergence of dormant DTCs. A study by Weidenfeld and colleagues demonstrated that LOXL2 expression induced by hypoxia in dormant breast DTCs promoted their epithelial to mesenchymal transition (EMT). This in turn endowed the cells with stem-like properties leading to their escape from tumor dormancy both *in vitro* and *in vivo*, while inhibiting LOXL2 expression prevented their outgrowth (96, 97). Overall, these studies suggest that LOX/LOXL2 may serve as a therapeutic target to prevent the emergence from tumor dormancy to overt metastases (**Figure 1**).

Interestingly, a recent report demonstrated how a systemic inflammatory response induced after surgery promotes the emergence of dormant immunogenic DTCs at distant anatomic sites while, preoperative anti-inflammatory treatment with meloxicam, a nonsteroidal anti-inflammatory drug (NSAID), prevented the outgrowth of DTCs in the lungs (74). Notably, these finding are in line with a retrospective analysis carried out on breast cancer patients who received anti-inflammatory analgesics prior to surgery. These patients exhibited reduced incidence of early metastatic relapse (98, 99).

Overall, the studies presented here emphasize the important role of inflammation and/or fibrosis in dormant DTCs outgrowth and also reinforce the notion that intervention in the perioperative stage and immediately after re-section may be critical to prevent local and/or distant recurrences.

## Eradicating Dormant DTCs Before They Awaken

Once DTCs anchor in their new and “non-permissive niche” adaptive cell-intrinsic mechanisms ensure their long-term survival and escape from immune surveillance (41, 42, 100). These hibernating cells resist most traditional and newer targeted agents given their quiescent state and/or their induced senescence–like state (101) or as recently suggested due to the BM perivascular niche (100). Hence, unraveling cell-intrinsic mechanisms responsible for long-term survival of DTCs along with the mechanisms that enable their chemoresistance and immune evasion may open up new approaches to eradicate these dormant DTCs.

### Targeting Cell-Intrinsic Mechanisms Mediating Long-Term Survival of DTCs at the Foreign Niche

The mechanisms responsible for the long-term survival of DTCs are just beginning to emerge.

**Figure 2:**
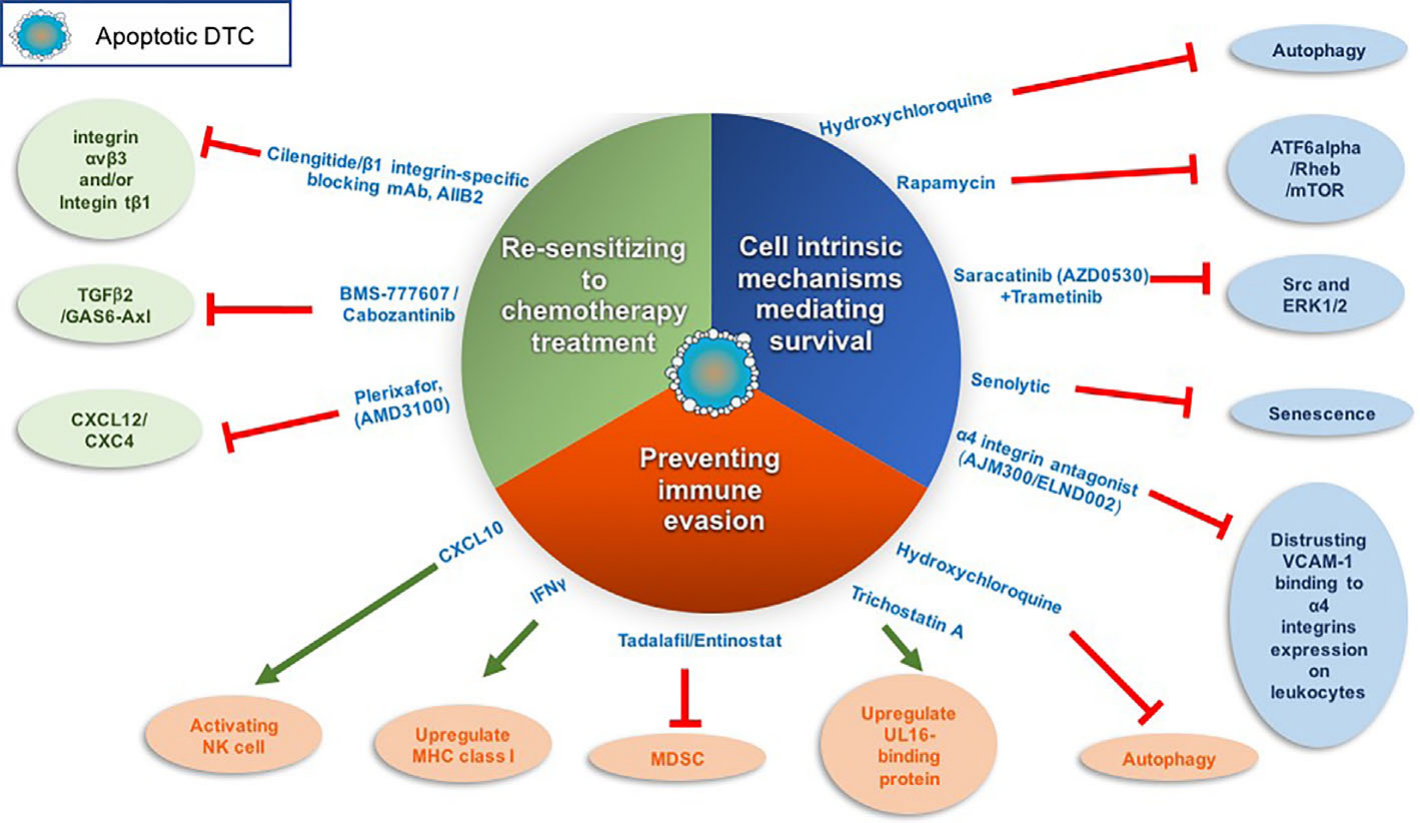
Targeting dormant DTCs for eradication. The following scheme illustrates the different strategies and corresponding drugs that we may utilize to eradicate dormant DTCs. These strategies include inhibiting cell-intrinsic mechanisms of dormant DTCs long-term survival, sensitizing dormant DTCs to chemotherapy treatment and/or preventing dormant DTCs immune evasion. Red line denotes inhibition and green arrow denotes activation.

Previously, Src and ERK1/2 activation were shown to be essential for the survival and outgrowth of dormant breast DTCs. By utilizing a 3D model system and *in vivo* model system to study tumor dormancy (75, 77, 78) it was shown that only combined inhibition of ERK1/2 and Src in dormant breast cells culminated in their eradication (102). These findings suggest that combining a Src inhibitor such as saracatinib (AZD0530) with the FDA-approved MEK1/2 inhibitor trametinib may eradicate dormant breast tumor cells before they awaken (**Figure 2**).

The activation of the transcription factor ATF6α was shown to regulate the survival of quiescent squamous carcinoma cells. ATF6α activation induced survival through the up-regulation of Rheb and activation of Akt-independent mTOR signaling (103). Of note, two mTOR inhibitors have been approved by the FDA to treat cancer and several are under clinical investigation as a combination or monotherapy. However, these clinical trials to date are designed to test the drug efficacy in advanced cancer or recurring cancer patients but not as a monotherapy to target dormant DTCs (104) (**Figure 2**).

Another intrinsic mechanism shown to regulate DTCs survival is autophagy (71, 105, 106) (**Figure 2**). Inhibition of autophagy in dormant breast and osteosarcoma cells by hydroxychloroquine promoted apoptosis of the dormant breast DTCs that have colonized the lungs and sensitized dormant osteosarcoma cells to cytotoxic anticancer agents (106, 107). Interestingly, autophagy was recently shown to restrict the outgrowth of micrometastases of several murine models of breast cancer while inhibition of autophagy lead to their outgrowth (108). Hence, inhibition of autophagy might differentially impact quiescent solitary dormant DTCs vs. micrometastases.

Notably, hydroxychloroquine is being widely used in cancer clinical trials in order to re-sensitize cancer cells to conventional therapies. Potential use of this drug as a preventive treatment to eradicate early DTCs may warrant further investigation (109). Currently, a Phase II trial of hydroxychloroquine with everolimus for prevention of recurrent breast cancer is ongoing, as well as an ongoing Phase Ib/II trial of gedatolisib (inhibitor of PI3K/mTOR pathway), hydroxychloroquine or combination of both [reviewed in (110)].

Aberrant expression of vascular cell adhesion molecule-1 (VCAM-1) on dormant breast DTCs was also found to ensure their survival once they colonize the lungs (**Figure 2**). Specifically, VCAM-1 promoted PI3K/Akt activation and cancer cell survival by its engagement with the counter-receptor α4 integrins expressed on leukocytes. Furthermore, antibodies against α4 integrins blocked pro-survival signals induced by VCAM-1 (111). Therefore, disrupting the interaction between VCAM-1 and α4 integrins may potentially serve as a therapeutic target to promote dormant DTC eradication (112). Such drugs are already in clinical trials for the treatment of relapsing multiple sclerosis (MS) and inflammatory bowel disease (IBD). Currently, an orally active α4 integrin antagonist, AJM300, is under clinical trials in IBD patient [reviewed in (112)].

Notably, senescence of tumor cells that have escaped cytotoxic therapy has been proposed by several groups as another form of cell-intrinsic mechanisms that confer tumor dormancy and survival of circulating tumor cells (CTCs) and/or DTCs residing at the perivascular niche [reviewed in (101)]. This emerging concept warrants further exploration as it may open up an interesting opportunity to target these senescent-like cells with senolytics (small molecule drugs with selective killing of senescent cells) (113, 114) (**Figure 2**). Indeed, several recent papers have demonstrated how senolytic drugs caused cell death of senescence induced cancer cells (115–118).

Overall, these promising studies highlight the importance in further investigating the cell- intrinsic mechanisms governing survival of dormant DTCs.

### Sensitizing Dormant DTCs to Chemotherapy Treatments

Signals arising at their foreign niche can induce cell-intrinsic mechanisms governing the survival of dormant DTCs and their escape from cytotoxic therapies. C-X-C motif chemokine ligand 12 (CXCL12) has been reported to constitute aspects of the dormant niche and act as a survival factor. CXCL12 secreted by osteoblasts was shown to induce the survival of disseminated breast tumor cells expressing the CXCR4 receptor by upregulating the Akt pathway *via* c-Src activation (119). Furthermore, the CXCL12/CXCR4 axis was shown also to mediate the localizing and tethering of prostate cancer cells and of breast cancer cells to the BM (120–122) and regulate their growth. Therefore, disrupting the CXCL12/CXCR4 axis may impinge on the survival of dormant breast tumor cells or induce the outgrowth of prostate tumor cells (123). Plerixafor (AMD3100), a CXCR4 antagonist approved by the FDA, was shown in a subcutaneous xenograft mouse model of human prostate carcinoma to dissociate the prostate cancer cells from their sanctuary site (the BM) and thus sensitize them to chemotherapy treatment (124). This approach was demonstrated to be effective also for other cancers, such as leukemia (125–127). Thus, dormant DTCs may be sensitized to chemotherapy by promoting their proliferation making them vulnerable to chemotherapy treatment (**Figure 2**).

F-box/WD repeat-containing protein 7 (FBXW7) was shown to promote quiescence by ubiquitylation and proteasomal degradation of cell cycle promoters. Ablation of FBXW7 in dormant breast cancer cells caused DTCs to exit their quiescent state, which sensitized the cells to paclitaxel treatments in mouse xenograft and allograft models (128).

Recently, the TGFβ2/GAS6-Axl axis (56) was shown to be necessary for the induction of dormancy of prostate cancer cells in the BM. Treatment of dormant multiple myeloma cells with inhibitors to Axl such as BMS-777607 or cabozantinib released the cells from the endosteal niche in the BM, causing their reactivation, thus suggesting these cells could be susceptible to chemotherapy treatment (129). Indeed, in previous studies in solid tumors, combining chemotherapy treatment with inhibitor of Axl reduced tumor burden (130).

Another protein mediating the engagement of cancer cells to the BM niche is osteopontin. Osteopontin is an ECM protein secreted by osteoblasts in the BM niche and was shown to anchor acute lymphoblastic leukemia (ALL) cells to the BM culminating in their dormancy. Whereas, inhibition of osteopontin promoted ALL escape from tumor dormancy and sensitized them to cell-cycle–dependent Ara-C chemotherapy (131).

Overall, these studies suggest that dissociating dormant DTCs from their ‘safe haven’ niche can promote the cells to exit their quiescent state and potentially sensitize them to chemotherapy treatments.

Importantly, there are striking similarities between dormant DTCs and quiescent normal hematopoietic stem cells (HSCs), which reside in the BM. These similarities are exhibited by intrinsic mechanisms for survival, quiescence and their mobilization to the BM [reviewed in (43, 132)]. Therefore, when considering a strategy to dissociate dormant DTCs from their sanctuary site in order to sensitize DTCs to cytotoxic chemotherapy treatment it is critical to be sure that this will not facilitate depletion of HSC or cause reawakening of chemoresistant DTCs.

A recent report by Ghajar and his colleagues (100) demonstrated that disrupting the interaction between chemoresistant DTCs with the perivascular niche (PVN) by inhibiting Intβ1 and/or integrin αvβ3 sensitized DTCs to chemotherapy without inducing DTC proliferation (**Figure 2**). However, 22% of mice still succumbed to bone metastases upon combining integrin β1 inhibition with adjuvant therapy in this study.

Hence, there exists a risk of DTCs mobilization from their dormant niche. Therefore, another strategy that we may contemplate to eradicate dormant DTCs is by preventing their immune evasion.

### Breaking Off Immune Evasion of Dormant DTCs

A recent report by Malladie and colleagues demonstrated that cancer cells selected from lung and breast cancer cell lines for their competence to establish latent metastasis (dubbed LCC), acquired quiescence with stem-like characteristics by expressing the Wnt inhibitor DKK1. The authors demonstrated that autocrine DKK1 helps disseminated LCC cells enter quiescence. Furthermore, these quiescent cells downregulated natural killer (NK) cell ligands leading to evasion of immune surveillance. Specifically, quiescent LCCs downregulated cell surface UL16-binding protein (ULBP) activators of NK cell-mediated cytotoxicity, as well as receptors for cell death signals. Therefore, quiescent (dormant) LCCs escaped cytotoxic killing by NK cells whereas proliferating LCCs were eradicated by activated NK cells (133). Of note, this study was conducted in immune-compromised mice and hence the role of NK cells in fully immune-competent animals remains to be determined.

Overall, these initial findings may open up in the future a novel therapeutic approach to selectively eradicate dormant DTCs by inhibiting their immune evasion. This will require re-expression of NK ligands in dormant DTCs. Interestingly, the inhibitor of histone deacetylases (HDACs) trichostatin A (TSA) was shown to induce ULBP expression in epithelial cancers (134). Whether TSA can inhibit the immune escape of dormant DTCs remains to be explored (**Figure 2**).

Notably, several studies demonstrated how autophagy in cancer cells can impair the susceptibility of the cancer cells to NK-mediated killing (135). Given that autophagy was shown previously to be launched in dormant DTCs (71, 106), it will be worth exploring whether inhibiting autophagy of dormant DTCs will not only impinge on their direct survival but may also enable cytotoxic killing by NK cells (**Figure 2**).

Another mechanism by which DTCs were shown to evade the immune system is by downregulation of the expression of major histocompatibility complex class I protein (MHC-I), thus evading CD8+ T cell recognition (136). Furthermore, the MHC-I negative phenotype of DTCs in the BM was shown to be associated with poor survival in curatively resected breast cancer patients without distant metastases (137). Similarly, single quiescent DTCs colonizing livers from patients and mice with pancreatic ductal adenocarcinoma (PDAC) were MHC-I-negative and exhibited unresolved endoplasmic reticulum (ER) stress. Notably, once these quiescent DTCs were resolved from their ER stress the cells emerged from their dormant state but also regained their MHC-I expression. Therefore, outgrowth of these cells occurred only when these quiescent DTCs were resolved from their ER stress and the T cell response was disrupted (138).

Hence, upregulating MHC-I may be an attractive approach to reinstate DTCs vulnerability to immune surveillance (**Figure 2**). Notably, epigenetic control mechanisms regulating MHC-I expression have been frequently detected [reviewed in (139)]. Furthermore, interferon γ (IFNγ) was shown by several studies to act as an epigenetic modifier upregulating the expression of antigen-presenting machinery genes such as MHC-I (140). Future studies should be pursued in order to study whether treatment of dormant quiescent PDAC/breast DTCs with IFNγ will induce MHC-I antigen expression and eradication by CD8+ T cells. This kind of approach needs to take into account: i) whether the dormant DTCs with MHC-I downregulation can be sensitized to IFN-γ treatment. Given that approximately 30% of human tumor cells exhibit reduced IFN-γ sensitivity as a result of an impaired expression in the different components of the IFN-γ signaling (139) and ii) the clinical stage and context by which this treatment will be applied. Considering that IFN-γ has pleotropic effects on different stages in tumor progression (141). In addition, IFN-γ also is toxic given systemically, so inducing expression locally is likely to be more successful than systemic administration.

Overexpression of immune checkpoint proteins on dormant tumor cells was also shown to facilitate their immune escape. Dormant tumor cells in the DA1-3b/C3H mouse model of AML evade cytotoxic T-lymphocyte (CTL)-mediated killing because they overexpress PD-L1 (B7-H1) and CD80 (B7-1) (142). PD-L1 binds to receptors on CTLs (PD-1) and thus promotes CTL death and exhaustion. Importantly, this immune evasion was overridden by activating NK cells with CXCL10 (143). Overall, these findings suggest that reinstating either MHC-I and/or NK cell ligands, as well as inhibiting immune checkpoints proteins on dormant DTCs, may re-sensitize them to cytotoxic killing by T cells and/or NK cells (**Figure 2**).

Myeloid–derived suppressor cells (MDSCs) may also indirectly regulate the survival of dormant DTCs. MDSCs represent a population of special cells of the immune system, which consist of immature macrophages, immature granulocytes, and immature dendritic cells. MDSCs suppress activation of T and NK cells through the production of reactive oxygen species (ROS) and arginase 1 (Arg-1), along with recruitment of other immune suppressive cells such as regulatory T cells [reviewed in (144)]. Accumulating evidence suggests that enrichment and activation of MDSCs correlates with cancer recurrence and poor clinical outcome.

Hence, modulating MDSC immunosuppressive activity may in turn prevent immune evasion of dormant DTCs. Inhibitors of phosphodiesterase-5, sildenafil and tadalafil, were shown to inhibit the immunosuppressive activity of MDSCs in preclinical and clinical studies by the downregulation of inducible nitric oxide synthase (iNOS) and Arg-1 activities (144). Promising results with tadalafil have been reported for head and neck squamous cell carcinoma and melanoma patients (145–147). Entinostat, a class I histone deacetylase inhibitor, was also shown to inhibit the immune suppressive activity of MDSCs [reviewed in (144)]. Importantly, entinostat has been evaluated in Phase I and II trials in patients with advanced malignancies, with a favorable risk–benefit profile [reviewed in (148)].

Overall, several drugs that have already been tested in the clinical setting may be considered for inhibiting the immune evasion of dormant DTCs thus potentially leading to their demise (**Figure 2**).

## Targeting Potential Gatekeepers in Regulation of Dormant DTCs and Their Supportive Microenvironment

Current studies which are just beginning to unravel the intricate crosstalk between the residing dormant DTCs and their niche, highlight the complexity and the need to rethink the design of future therapeutic strategies to prevent dormant DTCs from ever emerging. If a common multi-faceted target that will both inhibit dormant DTCs and their niche can be identified, the cross-talk between them could be affected leading to their long-term hibernation or their demise.

### Potential Gatekeepers That We May Consider Inhibiting Based on Current Studies Are STAT3 and Reinstating NR2F1 Expression

A potential multifaceted target that has been demonstrated to be a molecular hub in mediating tumor escape from immune surveillance (149) and regulate the outgrowth of dormant DTCs is STAT3. Expansion and immunosuppression of MDSCs is mediated by activation of STAT3 [reviewed in (149)]. Anti-inflammatory M2-like macrophages which also play an important role in metastasis at distant organs [reviewed in (150)] are polarized to the M2 phenotype by STAT3 activation [reviewed in (149)]. Furthermore, in addition to STAT3’s role in immune suppression, STAT3 activation was recently shown to also directly mediate dormant DTC outgrowth. DDR1 interaction with Col-I at the permissive niche induced non-canonical signaling converging on activation of STAT3 in dormant breast DTCs, which culminated in their outgrowth at multiple organ sites (92). Notably, in paraffin-embedded tissue microarray sections of human breast cancers there was a significant increase in the expression of phosphorylated STAT3 (pSTAT3) in lung metastases compared to their matched primary tumors and the highest levels of pSTAT3 were present in those that had recurred after a short disease-free interval (92). These results suggest a role of activated STAT3 in metastatic outgrowth of dormant breast DTCs in the lungs. Therefore, targeting STAT3 may prevent both dormant breast DTCs outgrowth at the permissive site in the lungs and the formation of an immune suppressive niche.

Hence, STAT3 may serve as an attractive clinical target given that inhibitors of STAT3 have already entered clinical trials along with newer inhibitors at the preclinical stage [reviewed in (151)]. Moreover, several FDA-approved drugs were shown already to inhibit STAT3 signaling and thus may be repurposed to potentially prevent the outgrowth of dormant breast DTCs (152). However, it is important to keep in mind STAT3’s central role in signaling networks and therefore targeting it may lead to toxicity.

Of note, STAT3’s role in cancer recurrence is just beginning to unravel, and a recent study in contrast demonstrated that dormant breast DTCs residing in the BM niche remain dormant upon activation of STAT3 *via* the activation of leukemia inhibitory factor (LIF) receptor (53). Furthermore, inhibition of STAT3 led to bone osteolysis resulting in the outgrowth of dormant DTCs (53). Hence, LIFR : STAT3 signaling appears to confer a dormancy phenotype in breast cancer cells disseminated to bone. In addition, STAT3 was shown to be part of a pro-dormancy gene signature for estrogen receptor positive breast tumors (153)

Therefore, further research needs to be conducted in order to clarify the role of STAT3 in dormancy and outgrowth. It may be that the outcome of targeting STAT3 may depend on breast cancer subtype, the site of hibernation of the dormant DTCs and the precise timing of such intervention.

Another gatekeeper that holds great promise is NR2F1. Reinstating NR2F1 expression in the BM may prevent dormant DTCs from awakening by promoting cell-intrinsic dormancy programs in prostate and/or HNSCC cells while also reinstating the dormancy niche in the BM by secretion of BMP7 and TGFβ2 (69, 70). Furthermore, SPARC, which is a target of NR2F1 (58), was shown to regulate tumor dormancy of prostate cancer cells by promoting the expression of BMP7 in BM stromal cells.

Indeed, recent studies demonstrated that combining the epigenetic regulating drug 5-AZA with the differentiation agent trans-retinoic acid (ATRA), reinstated in part NR2F1 expression (70), while 5-AZA by itself reinstated SPARC expression in bone DTCs (58). This combination is now part of an ongoing Phase II clinical trial (https://clinicaltrials.gov/ct2/show/NCT03572387) to study combined 5-AZA and ATRA treatment on top of standard of care in recurrent prostate cancer patients based on rising prostate-specific antigen (PSA) only.

Overall, these studies may open up novel avenues to maintain DTCs dormancy.

## Potential Markers That Will Enable Stratification of Patients at High Risk for Recurrence

Once a therapy is developed to either target dormant DTCs for destruction or prevent their emergence from dormancy, the question arises as to which patients are at sufficient risk to warrant a therapy in the absence of overt disease. The decisions by clinicians to treat patients using such therapies will largely depend on the risk/benefit potential for such therapies, as well as healthcare-associated costs. Assuming that novel treatments to prevent recurrence will involve risks and substantial costs, the ability to stratify patients and determine for which patients the treatment is likely to provide benefit would be extremely valuable. This is true for clinical trials and post approval.

Patient prognosis and stratification in order to inform treatment decisions is far from being a simple endeavor. Dormancy and emergence from dormancy are presumably determined by properties of individual dormant DTCs and of the milieu in which they have lodged, as well as other factors such as the patient’s immune system. These properties may determine the risk/timing of relapse. Currently, detecting dormant DTCs through tissue biopsy is limited to BM. Furthermore, it is difficult to find biomarkers related to dormant DTCs, or provide prognosis predictions *via* imaging. Fortunately, using innovative approaches, it seems like we are gaining ground. Various technologies that utilized liquid biopsies enable collection of surrogate markers. In addition, it may be possible to predict recurrence based on properties of cells in the primary tumor. Hence, identifying biomarkers in liquid biopsies and in primary tumors that will enable stratification of patients that are at high risk for recurrence, while remaining a great challenge, offers great hope.

Current literature provides evidence for the identification of such markers specifically in breast cancer patients. A recent retrospective cohort study in breast cancer patients with a five-year follow-up after diagnosis found differential expression within the recurring tumors. The differential expression of the proteins was also related to breast cancer subtype. For instance, TNBC patients who recurred had significantly higher expression of Snail protein in their primary tumors compared to those without recurrence, whereas Twist expression was significantly higher in primary tumors of estrogen receptor and progesterone receptor positive breast cancer patients who recurred compared to those without recurrence (154). Notably, both Snail and Twist are transcription factors modulating the epithelial to mesenchymal transition (EMT). Similarly, an increase in mRNA levels of LOXL2, shown previously to induce EMT of dormant DTCs (96, 97) was shown to be associated with a significant decrease in the relapse free survival (RFS) of patients with lymph node-negative breast cancers. Hence, patients with increased levels of LOXL2 mRNA have a higher risk recurrence (96). Whether LOXL2 protein expression in primary breast cancer biopsies can serve as a predictive marker for cancer recurrence and whether it is subtype dependent warrants further investigation. Furthermore, future studies may determine whether combining several EMT markers as predictors of breast cancer recurrence might yield more definitive stratification of patients who are at high risk of recurrence.

Importantly, a 21-gene recurrence-score assay (Oncotype DX, Genomic Health) provides prognostic value. This gene-expression assay is used to assess risk of disease recurrence in hormone receptor-positive, HER2-negative breast cancer patients and to guide decisions regarding adjuvant chemotherapy (155). The assay provides a Recurrence Score (RS), ranging from 0 to 100, indicating low risk (RS < 18), intermediate risk (RS 18–30), or high risk (RS ≥ 31) of disease recurrence. Intermediate risk patients were recently shown by the large prospective TAILORx trial to receive little benefit from chemotherapy in regard to recurrence, with a notable exception for younger patients (156). Hence, early breast cancer patients who are hormone receptor-positive (ER+), HER2-negative with either high or intermediate RS score may benefit in the future from treatments designed to target dormant DTCs.

Indeed, a recent study demonstrated stratification of dormant DTCs in the BM of breast cancer patients based on their NR2F1 expression (157). Importantly, the presence of DTCs in the BM of breast cancer patients was also evaluated previously as potential prognostic marker for breast cancer recurrence (158–160). Bjorn Naume and his colleagues also demonstrated that DTCs status in the BM can identify breast cancer patients who are at high risk of recurrence after receiving adjuvant chemotherapy (161). In addition, a recent study demonstrated that the presence of DTCs in the BM of patients prior to surgery is a significant predictor of late recurrences, particularly for reduced survival in postmenopausal women patients with ER+ disease, lymph node involvement, and large tumors (162). Hence, it warrants further investigation whether DTCs assessment in the BM of breast cancer patients could supplement primary tumor diagnostics such as Oncotype DX and thus may yield more definitive stratification of breast cancer patients who could benefit from preventive treatment.

Importantly, although some gene expression assays of the primary tumor can be used to determine both early and later recurrence and the efficacy of extended adjuvant endocrine therapy in ER+ breast cancer patients, the use of such assays is not recommended for guiding therapy beyond 5 years (163, 164). Hence, other approaches such as liquid biopsies are emerging as potential prognostic markers to enable stratification of patients who are at high risk for recurrence.

Liquid biopsies, which includes circulating tumor cells (CTCs), circulating cell-free tumor DNA (ctDNA) and extracellular vesicles (EVs), may hold great promise in clinical diagnosis. The presence of CTCs in the peripheral blood of patients after tumor resection denote the existence of minimal residual disease and may provide insight into the process of metastatic spread and enable real-time monitoring of disease progression and therapeutic response [reviewed in (165)]. Several meta-analyses have highlighted the prognostic value of CTCs in various cancers, including breast (166), pancreatic (167), lung (168), colorectal (169) and prostate cancer (170).

A recent study by Sparano and colleagues demonstrated the prognostic value of CTCs in predicting late recurrence of breast cancer patients. The presence of CTCs in peripheral blood samples of hormone receptor–positive breast cancer patients obtained approximately 5 years after diagnosis provided independent prognostic information for late clinical recurrence (171). CTCs positivity was associated with a 13.1-fold higher risk of recurrence. These findings may provide in the near future the premise to stratify patients who may benefit from treatment aimed to target dormant breast DTCs.

The prognostic value of CTCs was also illustrated in prostate cancer patients. A recent study demonstrated CTCs detection in patients who had undetectable PSA levels following radical prostatectomy. Furthermore, these patients with CTCs had an increased risk of biochemical recurrence (defined by an increase of PSA levels) (172). Hence, CTCs may provide future prognostic value to help identify patients after radicalprostatectomy who are at high risk for recurrence. Overall, CTCs are emerging as a potential prognostic marker that may help stratify patients at high risk for cancer recurrence. Whether CTCs are dormant and/or are shed from indolent micrometastases is yet to be explored.

ctDNA is an emerging exciting novel technology in monitoring cancer progression and may guide treatment. ctDNA was shown to be present in plasma samples of many types of tumors that had not apparently metastasized or released CTCs to the circulation (173). Importantly, the total amount of ctDNA at the early stage of cancer patients might be <0.01% of the total circulating cell-free DNA concentration. In a healthy person, the latter is mainly derived from apoptotic leukocytes (165). These extremely low concentrations of ctDNA are approached by several methodologies that rely on a single tumor-specific mutation or a limited panel of mutations known *a priori* to be present in the primary tumor based on previous genomic analysis of the primary tumor. Using this approach Garcia-Murillas and his colleagues performed mutation tracking in plasma DNA of early breast cancer patients receiving neoadjuvant chemotherapy. Detection of ctDNA and mutation tracking of several plasma samples after completion of apparently curative treatment predicted metastatic relapse with a median lead time of 7.9 months over clinical relapse (174). This preliminary study suggests that ctDNA detection may provide prognostic information. Hence, the potential use of CTCs and ctDNA as predictors of late recurrence warrants further investigation. Of note, CTCs and ctDNA are extremely rare. In contrast, extracellular vesicles (EVs) derived from tumor cells are very abundant in blood, are highly stable, and could be used as a source of new biomarkers for personalized diagnosis and prognosis (175). EVs is a global term referring to several different classes of secreted vesicles and includes exosomes, microvesicles, ectosomes, large oncosomes, exosome-like vesicles, and apoptotic vesicles (176). EVs content varies with the type and includes proteins, mRNA, miRNA, long noncoding RNA, circular RNA and DNA, which play a crucial role in regulating tumor growth, metastasis, and angiogenesis. EVs content has been reported to predict recurrence of head and neck and colon cancer after chemotherapy treatment [reviewed in (177)].

Notably, Lev and her colleagues conducted proteomic analysis by reverse phase protein array on EVs content derived from plasma of breast cancer patients. They identified potential markers that can predict the risk of breast cancer recurrence (178). One such marker was HSP70, shown previously to be associated with tumor recurrence (179).

Overall, these exciting emerging technologies such as liquid biopsies hold great promise in developing non-invasive approaches to monitor cancer progression and predict cancer recurrence.

## Overcoming the Challenges of Metastasis Drug Development-Designing Trials to Measure Clinically Meaningful Effects Driven by Targeting Dormant DTCs

The end goal for dormant DTC targeting agents is to prevent, delay or minimize recurrence in patients who present with a primary tumor. Another major goal is to prevent, delay or minimize further progression in patients who present with a recurrence. For example, preventing additional recurrences in a patient who has a resectable metastatic lesion at a distant site.

As outlined herein, metastases may arise from dormant DTCs which persist at distant sites, have bidirectional interactions with their microenvironment, avoid the immune system and are undetected by current diagnostic procedures. While the primary unmet need for most cancer patients is preventing metastatic recurrence at secondary sites, the progress made in this area is still limited. Most drug development efforts focus on shrinking existing primary or metastatic tumors in preclinical models, and later in the clinic, and most small molecule and monoclonal antibody drugs are advanced based on their ability to target rapidly dividing cells. The clinical expectation is that overt metastatic tumors will respond and demonstrate a regression of a measurable lesion, with a direct correlation with improved survival and quality of life. Agents that target only dormant DTCs are predicted to have no measurable activity against proliferating metastatic lesions or primary tumors. Traditional trial metrics of event free survival (EFS), progression free survival (PFS) and overall survival (OS) identify only agents able to control dormant DTCs when this effect also controls other malignant cell growth of a measurable lesion. It is possible that drugs that are effective in current clinical trial designs also have an activity against outbreaking dormant DTCs. Therefore, a new approach that better aligns unmet therapeutic needs with drug development efforts is required.

Practically, upfront trials, where patients who present with a primary tumor are treated with standard of care plus drug/s that can target dormant DTCs are more challenging than trials with patients that already have had metastatic recurrence. This is due to the fact that only a percentage of patient who present with a primary tumor will recur, and due to the fact that the time for a first recurrence can be anywhere from months to many years. As an example, if a trial design is based on EFS at two years, where about 40% of the patients are expected to have an event, the trials must be sufficiently powered to detect improved EFS in the subset of patients who entered the trial. These trials require larger number of patients and they require a longer time of treatment and observation compared to trials in patients who already had relapse. Furthermore, unless agents given to such patients have been specifically shown to eliminate DTCs, they will need to be continuously administered potentially for many years, which is often a challenge, especially when therapies given in conjunction with standard of care that includes chemotherapy. Therefore, it is also less likely that an open label study would be pursued; it is much more likely that a controlled study with a new agent on top of standard of care would be compared to standard of care alone. Implications for time to recruit patients and overall costs is very substantial compared to the option of an open label study. This is especially true given the challenges of identifying the dose that would be optimal for Phase II and III trials. Briefly, since a trial design that utilizes maximum tolerated dose to seek an effect on lesion size is not possible when targeting microscopic dormant DTCs, establishing a selected dose is quite challenging. This is described at more length in the study by Steward et al. (16).

Alternatively, trials in patients that already had a relapse are expected in many cancers to require fewer patients, be shorter in duration of treatment and recruitment time, and importantly be suitable for open label study designs versus controlled studies. As an example, if patients with a certain cancer with well documented historical data have an EFS of 20% at 8 months post-surgical removal (resulting in no measurable disease), a study of a novel agent targeting dormant DTCs with an objective of doubling EFS to 40% at 8 months would require several dozen patients only. An alternate approach could be to target cancers that have a high prevalence of delayed metastasis but that occurs over a manageable time frame. Examples include osteosarcoma, TNBC and melanoma, although the latter also can recur a decade or more later.

Therefore, a development plan in which efficacy of dormant DTCs targeting agents can be determined in smaller trials in patients that have already recurred followed by larger trials in upfront settings is more likely to have support by clinicians, advocates and industry. Drugs that demonstrate efficacy in patients that already recurred can then be tested in the upfront trial scenario, informed by safety, efficacy and PK/PD data from the previous trials.

### Novel Trial Endpoints

As noted herein, dormant DTCs are undetectable at distant sites and reside as single/small cellular quiescent cells and or as small indolent micrometastases. Until it becomes possible to directly (e.g., *via* imaging or biopsy), or indirectly (e.g., *via* a biomarker) detect and follow such dormant DTCs and indolent micrometastases, we must rely on traditional clinical end points to assess the activity of drugs that may target and eliminate or control dormant DTCs. Unfortunately, there is no established trial design that completely accomplishes this task.

This is because clinical trials have several flawed assumptions in the context of examining effects on minor populations (180). The first is that clinical trials consider a cancer to be a single entity. Growth of existing lesions or development of new lesions is unequivocally considered progressive disease and thus failure. The second assumption that limits detection of effects on minor populations such as DTCs follows: trial endpoints are indifferent to whether failure (new lesion growth) was due to the outgrowth of dormant DTCs or outgrowth of small tumors below the detection limits of modern imaging. Finally, there is currently no method in clinical trials to distinguish between preventing the outgrowth of small tumor populations associated with dormant DTCs vs eradicating dormant DTCs, since both of these phenotypes would be considered a complete response if accomplished for a long enough duration of follow up.

**Figure 3:**
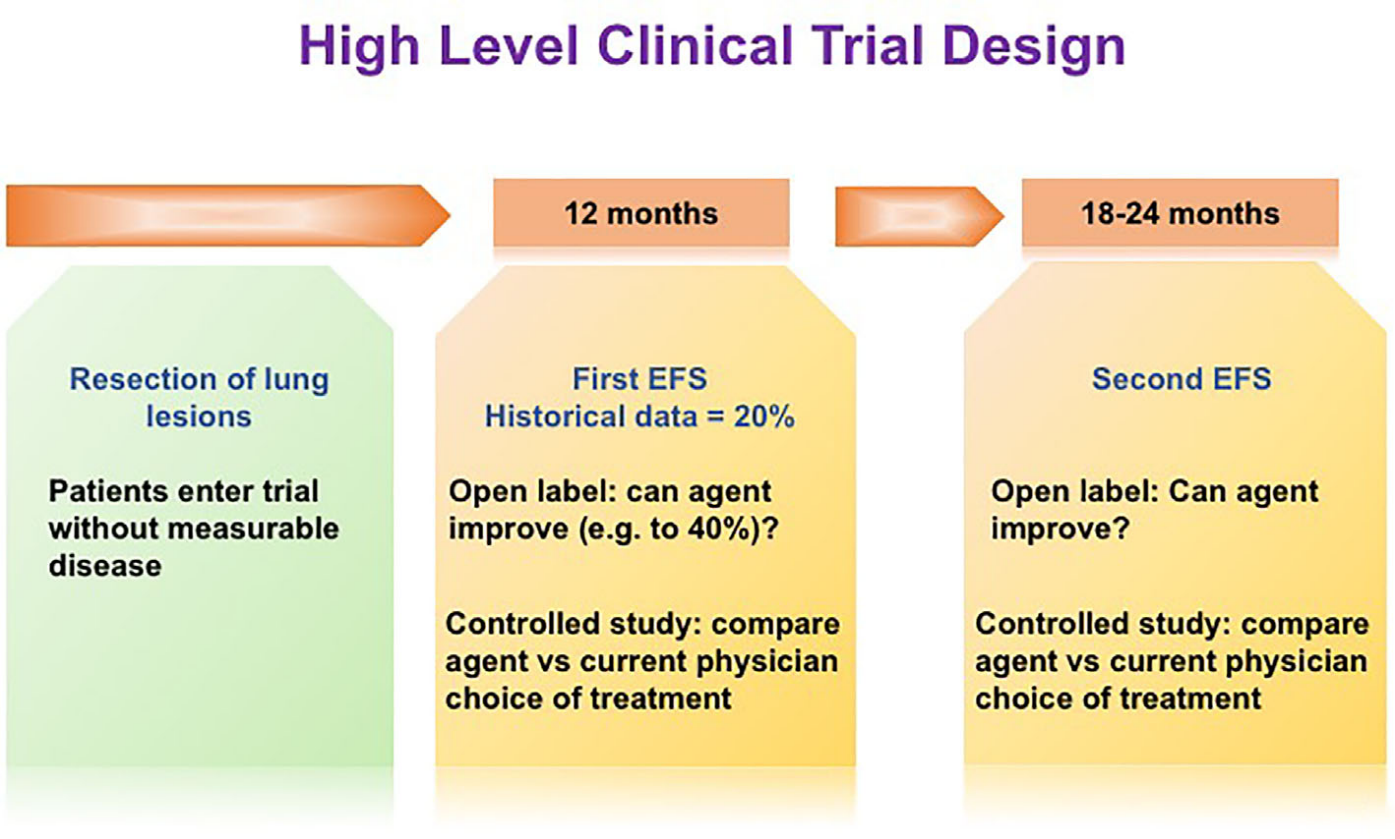
High level clinical trial design. In order to increase the ability to observe clinical impacts of novel dormant DTCs-targeting drugs, novel trial endpoints may be required. We propose an innovative trial design, namely, improving EFS at a later time point, e.g. 6-12 months following the initial EFS at 12 months post-surgery.

Herein lies the opportunity for novel trial endpoints to assess agents that affect dormant DTCs and contribute to improved outcomes. Osteosarcoma (OS) provides an excellent disease model for this discussion. OS recurrence is observed in about 40% of patients presenting with localized disease who are treated with standard of care, most commonly to the lungs within a few years of completing therapy (181). About 80% of patients presenting with metastatic disease and who achieve a second complete remission by surgical resection will have further additional recurrences in the lungs. It is unclear if systemic therapy provides benefit when recurrent disease is amenable to resection and thus there is not a standard of care for systemic therapy either before or after surgical resection of lung metastases (181, 182). Thus, the clinical trial community has adopted historically controlled trial designs for this population whereby the null hypothesis is that 20% of patients after resection of the lung lesion will remain without disease at 12 months and an active agent being defined by doubling this to 40% of patients remaining without detectable lung lesions at 12 months (183). (**Figure 3**). Controlled trials in which a therapeutic agent is compared to clinical choice can also be utilized in Phase II or in pivotal or post approval studies with the above time lines informed by historical data. While this trial design captures failure of both growth of subclinical cancer populations and recurrence from DTCs, it is unable to distinguish between these modes of failure.

**Figure 4:**
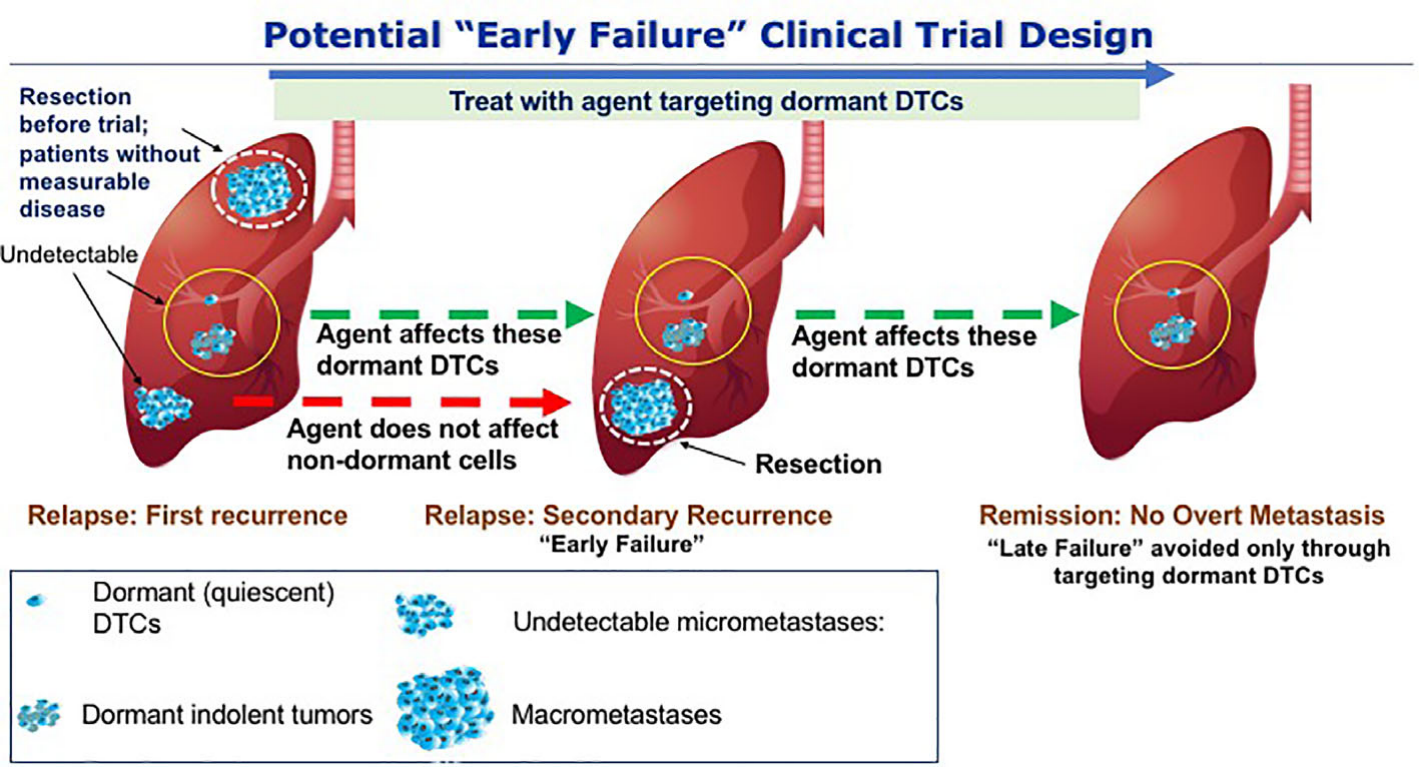
Potential trial to avoid late clinical failure through targeting dormant DTCs. The scenario presented here hypothetically addresses potential drugs that will prevent the outbreak of dormant DTCs in the lungs of OS patients. At the first event of relapse, dormant DTCs that are either quiescent and/or indolent are in the background of proliferating metastases. Upon resection and treatment with the drugs that targets dormant DTCs, cells that are not dormant will not be affected by the treatment and thus may lead to secondary recurrence (“early failure) and therefore a clinical benefit may only be evident at a later time point, i.e., following the initial “failure” to achieve a clinical outcome. By resecting the proliferating metastases and continuing the treatment we may avoid the outbreak of the residing dormant DTCs thus preventing “late failure”.

The hypothesis that we believe needs to be tested clinically involving dormant DTCs posits a limited number of dormant DTCs and/or dormant indolent tumors either residing alone or in the presence of undetectable micrometastases. Thus, in the presence of undetectable micrometastases even a 100% effective agent at either eliminating dormant DTCs or preventing their outgrowth may fail since the preformed micrometastases may progress clinically. Thus, in order to demonstrate the effectiveness of dormant DTCs targeting therapies in OS, novel trial designs are required. This study design would be based on the hypothesis that some undetectable tumors are too advanced to be affected by drugs that target dormant DTCs. In other words, if microscopic micrometastases resulting from outbreaking dormant DTCs are already present at the time of initial recurrence, they will emerge as additional lesions with or without DTCs treatment. Such a recurrence could be considered an “early failure” and its presence will not truly reflect the efficacy of an agent preventing the outgrowth of dormant DTCs or eliminating dormant DTCs (**Figure 4**). We thus propose an innovative trial design, namely, adding a primary aim of improving disease remission at a later time point (e.g., 2 years following first recurrence in addition to the initial EFS at 12 months post-surgery per point 2 above). This could be an open label study informed by historical data or a controlled study in osteosarcoma. In this scenario, an agent controlling or eliminating dormant DTCs, which would fail by any current trial methodology that removes patients from study at first progression (if any non-dormant cells are present), will have a chance to demonstrate important longer term disease control and clinical benefit missed by current trials.

## Future Directions

Metastatic recurrence is the major cause of mortality of cancer patients and poses a major unmet clinical therapeutic challenge. Unraveling the mechanisms underlying recurrence which arises years and decades following a protracted period of tumor dormancy may open up novel avenues to prevent disease from recurring. Being able to prevent delayed metastasis, either by specifically targeting dormant DTCs for destruction or maintaining them in the dormant state forever would represent a significant step forward and could save many lives. In this review, we introduced several potential strategies and drugs, some that can be repurposed and may prevent the outbreak of the hibernating DTCs based on growing studies in the field. These strategies aim to maintain the DTCs in indefinite dormancy and/or eradicate them. We also proposed to identify potential gatekeepers in regulation of dormant DTCs and their supportive microenvironment. These strategies could also potentially be complementary to each other. We have also provided various mechanisms and drugs, including some that may be repurposed with a shorter path to treating patients compared to novel compounds in preclinical stages. Notably, each strategy presented here has its complexities and must take into consideration the therapeutic window for treatment and patient stratification that would benefit from such treatment. Ideally, a preventive treatment should be started at the neoadjuvant/adjuvant setting to prevent the disease from ever emerging and/or prevent, delay, or minimize further progression in patients who present with a recurrence.

In order to increase the ability to observe clinical impacts of novel dormant DTC targeting drugs, novel trial endpoints may be required. Since novel dormant DTC targeting drugs are designed to target such DTCs before they exit dormancy, non-dormant cells are likely to progress during treatment and therefore a clinical benefit may only be evident at a later time point (i.e., following the initial “failure” to achieve a clinical outcome). However, later outcomes that would be expected by controlling the dormant DTCs with novel drugs may be expected and could have meaningful impacts on disease progression and survival.

Overall, these strategies could potentially open up novel avenues in the battle against cancer recurrence and may develop a strong foundation for developing drugs that would ensure that cancer will never recur.

## Author Contributions

SS, MI, RH, and DW: research, writing, and editing. DR: conception and writing. DB: conception, research, writing, and editing. All authors contributed to the article and approved the submitted version.

## Funding

DB supported by a Research Career Development Award from the Israel Cancer Research Fund, Israel Cancer Association, United States-Israel Binational Science Foundation grant # 2017237 and Israel Science Foundation grant # 942/20. DR supported by the National Pediatric Cancer Foundation (nationalpcf.org).

## Conflict of Interest

SS, RH and DW were employed by Vuja De Sciences Inc. DB and MI have consulting agreements with Vuja De Sciences, Inc.

The remaining author declares that the research was conducted in the absence of any commercial or financial relationships that could be construed as a potential conflict of interest.
